# Spurious Hyperchloremia and Negative Anion Gap in a Child with Refractory Epilepsy

**DOI:** 10.1155/2016/7015463

**Published:** 2016-02-11

**Authors:** Madhuradhar Chegondi, Balagangadhar R. Totapally

**Affiliations:** ^1^Division of Critical Care Medicine and Nemours Children's Hospital, Orlando, FL 32827, USA; ^2^University of Central Florida College of Medicine, Orlando, FL 32827, USA; ^3^Division of Critical Care Medicine and Nicklaus Children's Hospital, Miami, FL 33155, USA; ^4^Herbert Wertheim College of Medicine, Florida International University, Miami, FL 33199, USA

## Abstract

We report a case with spurious hyperchloremia with negative anion gap in a child who was taking potassium bromide for refractory epilepsy. Blood chemistry showed a high chloride level (171 mEq/L) and a negative anion gap (−52 mEq/L). Plasma chloride concentration is measured by an ion-selective electrode method; however the presence of other anions like bromide and iodides can interfere with chloride level and largely overestimates the chloride concentration. Thus hyperchloremia with a negative anion gap is a clue to the diagnosis of halides like bromide and iodide ingestion.

## 1. Introduction

Falsely elevated serum chloride level with negative anion gap is an uncommon occurrence and most likely due to laboratory error. A similar finding on consecutive electrolytes estimation suggests ingestion of medications containing halides such as bromide or iodide.

## 2. Case Presentation

A twelve-year-old girl, with past medical history of Dravet syndrome and developmental delay, presented to our hospital emergency department with altered sensorium, one-day history of fever up to 105.6 F, decreased oral intake and urine output, fatigue, and intermittent cough. Seizure frequency was unchanged. Her home medications were valproic acid, clobazam, potassium bromide, and diazepam. On arrival to the emergency room she was found to be hypotensive (65/39 mmHg), tachycardic, and febrile. She received two normal saline boluses. Her laboratory results in emergency room included serum sodium 143 mEq/L, potassium 4.3 mEq/L, chloride 171 mEq/L, bicarbonate 24 mEq/L, Blood Urea Nitrogen (BUN) 12 mg/dL, serum creatinine 0.57 mg/dL, calcium 9.2 mg/dL, blood glucose of 60 mg/dL, and valproic acid level 23.7 mcg/L (therapeutic level 50–100 mcg/L), and normal liver function tests were performed. Her capillary blood gas revealed pH 7.40, PCO_2_ 37.1 mmHg, bicarbonate 23.2 mEq/L, and base deficit −1 mEq/L. In the pediatric intensive care unit (PICU) patient remained stable, received intravenous fluids, was tested positive for influenza, and started on oseltamivir. Repeat electrolyte panel on multiple occasions, as shown in Table 1, had hyperchloremia (154–171 mEq/L) with negative anion gap (−40 to −52 mEq/L). Due to persistent hyperchloremia and as the patient takes potassium bromide for the past two years for refractory seizures possibility of bromide toxicity was considered. On day one of admission to PICU, potassium bromide therapy was stopped and serum bromide level was sent. The bromide level was 691 mcg/mL (normal therapeutic range 200–1000 mcg/mL). Despite normal bromide level in our patient potassium bromide was temporarily withheld and continued on IV hydration therapy. Over the next three days serum chloride and child's clinical condition improved.

## 3. Discussion

Dravet syndrome is a rare genetic encephalopathy with intractable epilepsy, also known as severe myoclonic or polymorphic epilepsy in infancy. Seizures in Dravet syndrome are resistant to treatment and require multiple anticonvulsant medications including bromide to control seizures [1]. Potassium bromide has sedative and anticonvulsant properties. It is been used as anticonvulsant medication especially in children with refractory seizures. The drug has very high bioavailability, and its half-life is 9–12 days [2]. It has a narrow therapeutic index and intoxication is known to occur even with therapeutic doses [2]. Other bromide-containing compounds, dextromethorphan bromide and pyridostigmine bromide, are also known to cause spurious hyperchloremia and negative anion gap [3, 4].

Bromide toxicity presents with confusion, lethargy, ataxia, tremor, cerebral edema, and other neuropsychiatric symptoms like acute psychosis and delirium [5]. Gastric intolerance, anorexia, weight loss, and skin lesions like acne forming dermatitis are also described as adverse effects [6]. Our index patient did not show any neuropsychiatric symptoms or skin lesions. It is postulated that at high levels bromide ion replaces the chloride in nerve transport mechanisms, stabilizes neuronal membrane, and impairs the nerve transmission [6].

Bromide is a negatively charged anion similar to chloride. Plasma chloride concentration can be measured by coulometry and colorimetric and ion-selective electrode methods. The presence of other anions like bromide and iodides can interfere with chloride level and largely overestimates the chloride concentration. The degree of chloride overestimation is lowest with coulometry method and highest with ion electrode method [7]. Each bromide ion reacts with more than three chloride ions. A serum bromide level of 5 mEq/L measures an additional 20 mEq/L of chloride ions [8]. This spurious hyperchloremia can also be seen with severe hyperlipidemia and salicylate poisoning [9, 10]. Specific assay with inductively coupled plasma mass spectrometry confirms the presence of bromide level. Apart from bromide ingestion, iodide, lithium intoxication, and monoclonal proteinemia can give rise to negative anion gap [11]. Treatment with aggressive rehydration and diuretic s improves the bromide toxicity and in refractory cases hemodialysis may be needed [7].

## 4. Conclusion

In our index patient, the systemic symptoms can be attributable to influenza infection. However, uncertain correlation between bromide level and toxic symptoms [7] and improvement of patient's sensorium and serum chloride level with ongoing hydration makes bromide toxicity a possible diagnosis. Bromide toxicity should be suspected in any child on bromide containing drug therapy presenting with neurological symptoms along with hyperchloremia and a negative anion gap.

## Figures and Tables

**Table 1:** This table shows serial electrolytes, anion gap, and calculated serum osmolality.

Serum parameter	Day of admission			
	Day 1	Day 2	Day 3	Day 4
Sodium mEq/L	143	143	136	138
Potassium mEq/L	4.2	4.7	4.4	4.4
Chloride mEq/L	171	162	154	154
Bicarbonate mEq/L	24	26	22	25
Glucose mg/dL	97	93	180	96
BUN mg/dL	12	3	3	4
Creatinine mg/dL	0.5	0.4	0.3	0.4
Calcium mg/dL	9.2	9.2	8.3	9
Anion gap mEq/L	−52	−43	−40	−41
Calculated osmolality mOsm/Kg	295	292	283	281
Bromide level mcg/mL	691			
